# Health worker perspectives of Smart Triage, a digital triaging platform for quality improvement at a referral hospital in Uganda: a qualitative analysis

**DOI:** 10.1186/s12887-022-03627-1

**Published:** 2022-10-13

**Authors:** Stefanie K Novakowski, Olive Kabajaasi, Mai-Lei Woo Kinshella, Yashodani Pillay, Teresa Johnson, Dustin Dunsmuir, Katija Pallot, Jessica Rigg, Nathan Kenya-Mugisha, Bernard Toliva Opar, J Mark Ansermino, Abner Tagoola, Niranjan Kissoon

**Affiliations:** 1 Department of Anesthesiology, Pharmacology & Therapeutics, University of British Columbia, Vancouver, BC Canada; 2 Centre for International Child Health, BC Children’s Hospital, Vancouver, BC Canada; 3 WALIMU, Kololo, Kampala Uganda; 4 Department of Obstetrics and Gynaecology, BC Children’s and Women’s Hospital, University of British Columbia, Vancouver, Canada; 5 Department of Pediatrics, Jinja Regional Referral Hospital, Jinja, Uganda; 6 Department of Pediatrics, University of British Columbia, Vancouver, BC Canada

**Keywords:** Child, Delivery of health care, Digital technology, Health personnel, Triage, Critical care, Sepsis, Telemedicine, Point-of-care systems, Quality improvement

## Abstract

**Background:**

Effective triage at hospitals can improve outcomes for children globally by helping identify and prioritize care for those most at-risk of death. Paper-based pediatric triage guidelines have been developed to support frontline health workers in low-resource settings, but these guidelines can be challenging to implement. Smart Triage is a digital triaging platform for quality improvement (QI) that aims to address this challenge. Smart Triage represents a major cultural and behavioural shift in terms of managing patients at health facilities in low-and middle-income countries. The purpose of this study is to understand user perspectives on the usability, feasibility, and acceptability of Smart Triage to inform ongoing and future implementation.

**Methods:**

This was a descriptive qualitative study comprising of face-to-face interviews with health workers (n = 15) at a regional referral hospital in Eastern Uganda, conducted as a sub-study of a larger clinical trial to evaluate Smart Triage (NCT04304235). Thematic analysis was used to assess the usability, feasibility, and acceptability of the platform, focusing on its use in stratifying and prioritizing patients according to their risk and informing QI initiatives implemented by health workers.

**Results:**

With appropriate training and experience, health workers found most features of Smart Triage usable and feasible to implement, and reported the platform was acceptable due to its positive impact on reducing the time to treatment for emergency pediatric cases and its use in informing QI initiatives within the pediatric ward. Several factors that reduced the feasibility and acceptability were identified, including high staff turnover, a lack of medical supplies at the hospital, and challenges with staff attitudes.

**Conclusion:**

Health workers can use the Smart Triage digital triaging platform to identify and prioritize care for severely ill children and improve quality of care at health facilities in low-resource settings. Future innovation is needed to address identified feasibility and acceptability challenges; however, this platform could potentially address some of the challenges to implementing current paper-based systems.

**Supplementary Information:**

The online version contains supplementary material available at 10.1186/s12887-022-03627-1.

## Background

Identifying and prioritizing care for severely ill patients is an important step towards improving child health globally. This approach has the greatest potential for impact in low-resourced facilities with high patient attendance. In recognition of this, the World Health Organization created the Emergency Triage Assessment and Treatment (ETAT) guidelines [1] aimed at identifying and managing severely ill children in low-resource contexts [2]. These guidelines can improve clinical outcomes among pediatric patients when successfully implemented [3, 4]. To date, these guidelines have primarily been implemented using paper-based systems. Due to their complexity and potential for differing interpretations, routine implementation requires extensive training and memorization. This is impractical in environments with high staff turnover and patient burdens [5, 6]. Consequently, care is provided on a first-come, first-serve basis in most low- and middle-income countries (LMICs), resulting in suboptimal care and poor outcomes. Smart Triage is a digital triaging platform for quality improvement (QI) that aims to address this challenge [7].

Smart Triage includes a mobile triage application, a Bluetooth patient and treatment tracking system, and clinical dashboard. The platform is currently undergoing clinical evaluation at public health facilities in Uganda and Kenya [7]. The mobile application integrates pulse oximetry [8], a respiratory rate counter [9], and a parsimonious risk prediction algorithm developed using clinical variables, anthropometrics, and outcomes collected from patients presenting to local outpatient/emergency departments (OPD). The predictive algorithm uses danger signs found in ETAT to identify children in need of immediate care and a predictive risk model to stratify all other children as non-urgent, priority, or emergency [7, 10]. The child’s risk category, location, and wait time are displayed in real time through the clinical dashboard on screens, computers and tablets that can be viewed by hospital staff and patients. This approach aims to deliver prioritized care to trauma patients and critically ill children who typically present to the OPD with severe infection and sepsis, as infectious diseases such as pneumonia, diarrhoea, and malaria remain leading causes of death for children under 5 years globally [11]. The platform can also provide health workers and hospital administrators with analytics to support data-driven QI initiatives.

Smart Triage represents a major cultural and behavioural change in terms of patient management at higher-level health facilities at LMICs. To date, there has been limited implementation of digital platforms that collect real-time, standardized data to support triaging and QI within these countries. Understanding user perspectives is critical to the acceptability, sustainability, and future scale-up of Smart Triage, and can inform ongoing and future implementation of Smart Triage. The purpose of this study was to assess health workers’ perspectives of the usability, feasibility, and acceptability of Smart Triage at a regional referral hospital in Eastern Uganda where Smart Triage has been implemented.

## Methods

### Study design

We conducted a descriptive qualitative study informed by a phenomenology approach, which focused on describing the lived experiences and understanding the meanings health workers attributed to their experiences with Smart Triage [12, 13]. The study comprised of face-to-face interviews conducted in November 2021, as a sub-study of a larger clinical trial to evaluate Smart Triage (NCT04304235) [7]. Health workers were invited to retrospectively recall experiences and share perspectives on the usability, feasibility, and acceptability of the platform, focusing on its use in stratifying and prioritizing patients according to their risk and informing QI initiatives. For this study, we defined usability as the design factors that influenced the ability to use the platform and feasibility as the infrastructure requirements or operational factors that influenced the ability to use the platform. We defined acceptability as factors that influenced willingness to use the platform. The study is reported based on the Consolidated Criteria for Reporting Qualitative Research (See Additional File 1) [14].

### Digital triaging platform

All children presenting to the OPD with an acute illness were triaged by health workers using the Smart Triage mobile application, and stratified as emergency, priority, or non-urgent. Bluetooth Low Energy (BLE) beacons with a diameter of 3.5 cm and weight of 7 g were attached to colour coded lanyards corresponding to the patients’ triage category (emergency: red, priority: yellow, non-urgent: green). Each beacon was labelled with a unique identification number. BLE readers installed on the walls monitored a patient’s location by detecting the beacons as they were carried by a parent or child. To track when specific treatments were administered, designated treatment beacons (i.e., for intravenous (IV) antibiotics, IV fluids) were pressed by health workers in conjunction with pressing the patient beacons. This trigger was registered by the BLE readers installed in the treatment room. Health workers could also record a patient’s admission status in the clinical dashboard. All this information was displayed in real time through the clinical dashboard on computers and tablets that could be viewed by health workers. This information was also displayed through a public dashboard on screens that could be viewed by patients, except the public dashboard did not display the patient name. Instead, patients could identify themselves based on their beacon identification number.

Following implementation of the Smart Triage platform, health workers received training on how to use the platform to inform QI initiatives on identifying and prioritizing care for children identified as emergency cases. A QI team of 8–10 health workers from the OPD, in-patient ward, nutrition ward, pharmacy, laboratory, and medical records office was formed. The team met monthly to identify QI targets, develop solutions, and monitor progress using interactive Plan-Do-Study-Act cycles [15] informed by data collected by Smart Triage. Solutions were designed to influence the actions of all health workers involved in identifying and prioritizing care for critically ill children within the hospital. The clinical dashboard was used to generate weekly customized reports with data on progress measures identified by the QI team. These reports were displayed on hospital notice boards and available for review by all health workers.

### Study site

The study was conducted in the busy OPD of Jinja Regional Referral Hospital in Eastern Uganda. The pediatric ward admits on average 7,420 patients per year and the OPD sees 30 to 130 patients per day, with fewer children presenting on weekends and a higher number of children presenting on clinic days (i.e., for regular follow-ups of children with chronic conditions). From April 2021 to December 2021, Smart Triage was used as the clinical standard for triaging children in the OPD. Through repeated continuing medical events and peer-to-peer mentorship, 55 certified health workers and 33 medical students and nursing students in the pediatric ward were trained on the use of Smart Triage during this time.

### Data collection

We employed a purposive sampling approach to select 15 certified health workers representing various departments and cadres using Smart Triage, through consultations with hospital leadership and senior hospital staff within the pediatric ward. Participants included a pharmacy dispenser, medical records officer, community linkage facilitators, nursing officers, clinical officers, and doctors from the pediatric OPD, general in-patient ward, and nutritional ward. Community linkage facilitators help identify, educate, and counsel patients living with chronic illnesses, and coordinate referrals to appropriate care supports. Medical interns and nursing students were not recruited due to an ongoing medical interns’ strike at the time of recruitment and the study team’s decision to prioritize recruitment across all departments using Smart Triage, rather than all levels of health workers using Smart Triage. A sample size of 15 was estimated to be sufficient to reach thematic saturation based on the number of health workers currently using Smart Triage and categories of health workers included in the interviews. The study team determined that data saturation had been reached following the initial coding process, based on the limited frequency at which new themes and sub-themes emerged from transcripts reviewed near the end of the coding process.

Participants were approached in-person by a member of the study team with no personal relationship to the participants and who was not previously involved in implementing Smart Triage. After study participants gave consent, interviews were conducted by a female Ugandan research assistant (OK; MA Sociology) with experience conducting interviews with health workers and patients in hospital settings, using a semi-structured interview guide developed for this study (see Additional File 2). Field notes were taken during the 30 to 45-min interviews. The interviews were conducted in November and December 2021 in the nutrition or shared OPD staff offices located in the pediatric ward, depending on the participant’s role and the availability of the office for private or semi-private discussion. Interviews were conducted in English, the official language of Uganda, and recorded digitally with an audio recorder. No repeat interviews were conducted.

### Data analysis

Interviews were transcribed verbatim and analyzed using NVivo 12 (QSR International, Melbourne, Australia) following a thematic approach for identifying, analyzing and reporting themes [16]. A coding framework was developed deductively from the study objectives to cover factors impacting feasibility, usability, and acceptability (three major themes), and inductively to identify emerging sub-themes within each of these categories. The coding framework was developed in consensus by the study team (SN, OK, YP, DD, KP, JR, JMA, MWK). Three researchers (SN, JR, KP) transcribed interviews; and SN coded transcripts and collated major themes and sub-themes to generate an initial coding framework with guidance from OK and MWK. The study team met to refine the coding framework until no new themes emerged. Confidentiality was maintained by limiting access of study materials to authorized personnel and ensuring that no identifying information was included in the analysis.

### Trustworthiness

Trustworthiness was assessed based on credibility, dependability, confirmability, and transferability, as defined by Lincoln and Guba [17, 18]. To ensure credibility, themes and sub-themes were verified against the field notes of the research assistant who completed the interviews and minutes from QI meetings held by hospital staff during the implementation period (data triangulation) and a summary of findings was returned to one study participant for feedback (member checking). Our study team included researcher-clinicians (AT, NKM, BO, JMA, NK) with > 15 years clinical experience working at public hospitals in Uganda and implementing digital health and quality improvement initiatives at public and private-not-for-profit health facilities across sub-Saharan Africa and experienced qualitative health researchers who had no prior involvement in Smart Triage (OK, MWK). The study team helped to ensure dependability and confirmability by maintaining detailed notes throughout the research process, participating in regular discussions to review the research process and study findings, using the COREQ checklist (see Appendix File 1) to guide reporting of study details, and including representative quotes from study participants in the manuscript. To ensure readers have sufficient information to assess the transferability of our results, we have included detailed information about the setting, sampling, interview procedure (including interview guide), and characteristics of our sample.

### Ethical considerations

Ethics approvals for the parent clinical trial and this sub-study were obtained from the Makerere University School of Public Health Higher Degrees Research and Ethics Committee (IRB00011353, Study Protocol Number 743), Uganda National Council for Science and Technology (ID: HS528ES), and Children’s and Women’s Health Centre of British Columbia Research Ethics Board (H2-20-00484). All methods were performed in accordance with the relevant guidelines and regulations. Written informed consent was obtained from all participants.

## Results

A total of 17 potential health workers were invited; of these, 15 agreed to participate and two potential participants declined citing scheduling conflicts. Participants included 3 linkage facilitators from the OPD, 4 nurses (1 from the OPD, 2 in general care, and 1 from the nutritional ward), 3 clinical officers from the OPD, 3 doctors (including 2 pediatricians, with 1 from the nutritional ward), 1 pharmacist, and 1 medical records officer. All participants received QI training, and took part in at least one QI team meeting. Of these, 60% identified as women, and 6 were between 20 and 30 years of age, 2 were 30–40 years of age, 2 were 40–50 years of age, and 5 were over 50 years of age. All participants had completed post-secondary education. The median length of time since completing their first postsecondary education was 8 years (< 1 to 32 years). Participants had a median of 3 years’ experience working at study site (< 1 to 31 years).

Information shared by health workers was organized into three major themes: factors impacting usability, factors impacting feasibility, and factors impacting acceptability. Sub-themes identified within each of the major themes are summarized in Table 1.

**Table 1:** Factors impacting usability, feasibility, and acceptability of Smart Triage

Major themes	Factors (sub-themes) identified within each major theme
**Usability**	• Digital risk prediction algorithm • Color-coded lanyards • Improved patient tracking • Computer literacy • Equipment challenges
**Feasibility**	• Staffing and training challenges • Lack of supplies
**Acceptability**	• Impact on quality of care • Training on QI processes • Staff attitude challenges • Caregiver acceptability

### Factors impacting usability

#### Digital risk prediction algorithm

Health workers commented on how the mobile application’s built-in risk prediction algorithm for stratifying children according to severity of illness allowed them to more accurately identify patients who needed emergency care but did not show obvious signs of distress (e.g., heavily bleeding, unconscious). The algorithm uses vital sign measurements as well as ETAT danger signs to assign a risk category to a patient. One pediatrician noted: *“This digital approach helped us a lot to pick those who are not overt, those are occult, the cases of children who come and are not obviously physically seen as in danger.” [Participant 13, general care].* As a result, junior staff (e.g., linkage facilitators, junior nurses, and clinical officers) described being more confident in reporting emergency cases identified using the application to their seniors for immediate treatment. As one clinical officer highlighted:

*“Now, with these vitals, you can all know that according to our triage system, a patient with this and this is not okay. And you can even alert your senior with confidence, which wasn’t there.” [Participant 14, OPD].*

Health workers also spoke to how the data collected during triage (e.g., vital signs, anthropometric measurements) could guide treatment decisions after a child was admitted. A nurse described this:

*“I feel it is very important to us because for us, as we are looking at the dashboard, we are seeing that this patient is coming with this type of severe malnutrition. So, we get prepared to that particular type of malnutrition*… *For example, the glucose which we are going to give, we get ready with the milk.*” *[Participant 9, nutrition ward].*

#### Colour-coded lanyards and patient tracking

Health workers reported that the colour-coded system of red for emergencies, yellow for priority and green for non-urgent was a common language that staff and patients understood. This improved how health workers managed patients, including the identification and prioritization of emergency cases. As one clinical officer described: *“It has helped in a way that if I see a child putting on a red lanyard, even if I have someone there with yellow, I will have to tell them, “First order, because I need to send this one as fast as possible, you know. So it has helped me to attend to emergencies better as compared to before.” [Participant 12, OPD].*

Health workers were able to view a patient’s risk category and location on the clinical dashboard. This allowed them to quickly identify when emergency cases were not receiving treatment, and when they were lost within the facility. This led to behavioural change among health workers, as they would actively search for lost patients, and escort them between departments to prevent them from remaining lost. As one pediatrician noted: *“Initially, the mother would be triaged in the…outpatient and then they say “you take that corridor and there you turn” but now someone is able to escort. That escorting is very critical because the child will not get lost in the system and lose their time [to receiving treatment].” [Participant 8, nutrition ward].*

#### Computer literacy

Health workers identified pre-existing computer literacy as the major factor affecting how quickly they learned to use the platform. Health workers who had prior experience using smart phones were mainly those who were relatively younger and found it easier to use the app, compared to the less-experienced, typically older staff. A clinical officer described the low computer literacy as a challenge to using the dashboard and mobile application:

*“At first, some of our colleagues knew how to use computers and it was easy for them…But for us, these buttons on these local phones, they’ll tell you even spacing a word is hard. Getting the letters on the board, it was hard.” [Participant 14, OPD].*

Over time, the system because easier to use. One clinical officer described it as “*a walkover*”, but only “*with time, or with practice, [and] through experience.” [Participant 12, OPD].*

#### Equipment challenges

Health workers reported challenges in using the different equipment on children. For instance, the beacons used to track patient treatments and location were not easy to press, as one of pharmacist explained: *“The beacons, they tend to be… that long pressing bit of it. Sometimes they tend to refuse (to trigger the BLE reader).” [Participant 10, pharmacy].* As a result, health workers did not always enter treatments into the system and time to treatments were not recorded for all patients. In addition, pulse oximeters were difficult to use on newborns or when the child was restless or very cold. This led to staff spending more time trying to measure the correct vital signs, increasing time performing triage and causing delays in identifying and treating emergency cases that did not display any of the ETAT danger signs incorporated into the digital risk prediction algorithm. A linkage officer described this impact on the triage process: *“[Triaging] an emergency person is supposed to finish in 5 minutes. [But] you cannot skip any…part, you have to fill everything. You see the kid is dying, crying in pain, but the sensor refuse to read.” [Participant 5, OPD].*

### Factors impacting feasibility

#### Staffing and training challenges

Health workers identified human resource challenges as a major barrier to implementing the platform. Staff were not available to triage patients in the OPD during the evening or weekends. Patients admitted during these periods were typically only entered into the hospital’s paper-based records and not the digital triaging platform. One clinical officer spoke of these challenges when asked what they did not like about the system: *“The aspect where the clinician is required to triage by himself, then after triaging the patients they have to go ahead and clerk now manually in the book. That one has been a bit hectic but it is only on special duties, maybe weekends and evening duties, because mostly OPD runs from the morning to maybe 3 o’clock. So, we don’t have a team of triage that stays after, but remember patients still come in.*” *[Participant 12, OPD].*

Shifting of health workers to different wards and departments and the high intake of students in the OPD contributed to the need for constant on-the-job training and mentoring. This led to inaccurate data entry and the need for clinical officers to repeat vital sign measurements during clinical assessments. One clinical officer described this challenge: *“These students who come here are trained little time, they don’t understand these things. I saw one fidgeting with a SpO2 meter…I tell her, ‘You wrote this? When you look at this baby, do you think this respiratory rate is right?’ So they need to train these [students] better.” [Participant 7, OPD].*

However, health workers also had suggestions on how to address this issue in the future. One clinical officer spoke of the importance of having mentors within the hospital who could continuously train staff on triaging and use of Smart Triage: *“As much as the app is there we might get new people, we also need to be reminded regularly so that we don’t… end up with emergencies everywhere when some are not.” [Participant 12, OPD].*

#### Lack of supplies

Health workers also reported lack of supplies (e.g., drugs, syringes, blood) at the facility. Caregivers are sometimes required to purchase supplies from outside the facility, leading to delays in time to treatment that could not be overcome with Smart Triage. As one clinical officer described: *“Our only challenge maybe, is not with the app, but the system. In as much as you may identify an emergency and take them to where they are supposed to be, due to drug stock outs… you have to send the caregiver now to go and buy from outside. Sometimes they have come alone, sometimes they are alien in the place, for instance the referral from Luuka, so they do not know Jinja. It takes a bit of a longer time to give the first treatment because of just the drug stock out.” [Participant 12, OPD].* Lack of these supplies was reported to reduce staff motivation when using the new system, as described by a pediatrician: “*It becomes very demotivating when you, using the Smart Triage, you know you have identified an emergency and you can’t intervene in time. So, the frustrations are mainly with a weak health system platform.” [Participant 13, general care].*

### Factors impacting acceptability

#### Impact on quality of care

Health workers identified the capacity of Smart Triage to improve quality of care in their facility as a major acceptability factor of the system. Health workers across all levels of experience described how the mobile application helped them identify and prioritize care for emergency cases, leading to more timely care and improving patient outcomes. As one clinical officer described: *“Those who are bad off are picked first to reduce on death… [Smart Triage] helps us to pick them, and they’re pushed to where they are supposed to be before they deteriorate.” [Participant 14, OPD].* The purpose of Smart Triage was to improve time to treatment with an appropriate bundle of care for children identified as emergency cases. However, one pediatrician noted that pediatric mortality also declined after implementing Smart Triage: *“One of the things that wasn’t part of our key outcomes, during the period we started implementing… using other data sources, we recognized a reduction in mortality within the first 24 hours.” [Participant 13, general care].*

#### Training on QI processes

Establishing a QI team and providing training on QI processes was another acceptability factor because it motivated health workers across all levels of experience and departments to take on tasks that allowed them to meet the QI targets set during iterative Plan-Do-Study-Act cycles. When Smart Triage was implemented, health workers were trained by the study team on how to use the information provided by the platform to identify barriers to reducing time to treatment, develop potential solutions, and monitor the effect of these solutions. A clinician described how this approach differed from previous QI initiatives that focused on auditing compliance with specific procedures and availability of equipment or supplies, rather than actionable items to improve care: *“It wasn’t like…we are now going to maybe get a high grade, maybe [place as] the first ward. [Now] we know there is a challenge. Which really looked different from the previous [QI initiative].”[Participant 15, in-patient ward].* One pharmacist spoke to how they now used the dashboard to improve quality of care within his department: *“I normally look at it to see whether there is any client waiting… If I find that there is one, I am in pharmacy, but I get concerned. Then now I ask myself is it, is it the problem with my department or is someone, is someone in another department, maybe lab, maybe in emergency, or at the clinician’s table. So I find out and follow up and try to talk to every person so we can have that patient receive the service.” [Participant 10, pharmacy].*

The platform also allowed health workers on the QI team to identify where delays in receiving treatments were occurring, and identify solutions that facilitated more coordinated and timely care. For example, as one doctor noted, “*Pharmacists started listing the things to which they have. A drug list. So at least that one also helps when you are prescribing.” [Participant 15, in-patient ward].* As a result, clinical officers and doctors were less likely to prescribe a drug that was out-of-stock, reducing delays caused by caregivers needing to purchase supplies from outside the facility.

Health workers also spoke to the importance of training hospital staff who could lead future QI initiatives to support the sustainability of the platform. As one pediatrician asked, “*How do we ensure that we carry on these [QI] initiatives, as, as a facility? Are you going to have us have champions who are going to say “Oh, for me, I am going on to take on, champion, this [QI] initiative and it goes on?” [Participant 8, nutrition ward].*

#### Staffing attitude challenges

Health workers spoke to how some staff were resistant to use the new system because they perceived it as extra work, and staff who were not using Smart Triage believed that nurses and linkage facilitators who were involved in triaging patients were receiving extra pay for this work. For instance, the beacons used for patient and treatment tracking require regular cleaning, as one medical records officer described: *“This is how you, you check if a beacon has had saliva into it, because there was a common issues where kids put beacons in their mouth.” [Participant 7, records department].* Further, one clinical officer described how they helped a nurse triage patients on one occasion, but did not want to help in the future because, “*She will take it for granted…I will not do her work.” [Participant 7, OPD].*

However, staff attitudes towards Smart Triage changed and improved over time for some health workers, once the impacts of the platform on the quality of care for children became more evident to the health workers. One pediatrician described this overall change: *“At the end of the day… the objectives of the Smart Triage and the extra effort that has been put in, has been considered justified by the health workers. So at the end of the day, I think with time, the health workers have actually appreciated what it takes and what it means to use Smart Triage.” [Participant 13, general care].* A clinical officer within the OPD described why he started to appreciate Smart Triage: *“There is a satisfaction you get when you are offering a service to a patient, as opposed to before, where it was just clear, clear the line, clear the line.” [Participant 12, OPD].* A medical records officer also described how health workers’ hoped Smart Triage could be integrated with the hospital’s existing Health Management Information System (HMIS), indicating that perceived approval of Smart Triage by the Ministry of Health was important for improving staff attitudes towards Smart Triage: “*I think the execution was really good, I just wished it was the Ministry backing it… there’s hopes for that (integration with the Ministry’s HMIS) to happen.” [Participant 11, medical records office].*

#### Caregiver acceptability

Healthcare workers also described the caregivers’ reactions to the system. A nursing officer noted that caregivers of children who were identified as emergency cases were “*happy, because they don’t even sit and wait.*” *[Participant 3, general care].* Further, one nursing officer described how caregivers used “*to quarrel outside there (in the OPD waiting area), as if it is a market,” [Participant 9, nutrition department]* but this was less likely to happen because caregivers were more empathetic towards ‘red’ or emergency cases. However, health workers also noted that caregivers of children who were categorized as non-urgent were typically not seen until later in the day, leading to longer wait times for these children. A clinical officer summarized this: *“The one in red will enjoy. The one in green will not enjoy.” [Participant 14, OPD].*

## Discussion

The study assessed health worker perspectives of the usability, feasibility, and acceptability of Smart Triage, a digital triaging platform implemented at a referral hospital in Uganda. The platform incorporates a risk prediction algorithm, automated patient and treatment tracking system, and clinical dashboard to support health workers with identifying and prioritizing care for critically ill children. Health workers found most features of the platform usable, and reported that the platform was acceptable due to its positive impact on patient care and use in informing QI initiatives. High staff turnover and a lack of medical supplies at the hospital were the primary barriers to the feasibility of Smart Triage, while staff attitude challenges were the primary barrier to the acceptability of the platform.

### Implications for clinical care

Information and communication technology (ICT) and digital health platforms are becoming increasingly prevalent in LMICs [19, 20]. This expansion has been driven by increased mobile phone use and internet access, growth in commercial ICT markets, prioritization in national and global digital health strategies, and an increasing number of resources on technology selection, implementation guidance, and evidence of impact [19]. The COVID-19 pandemic further accelerated this expansion [20, 21]. There is the opportunity to leverage these platforms and technologies to support real-time data-sharing to drive QI and policy change in all areas of health, including in pediatric critical care [22].

Compared to paper-based implementations of ETAT and similar guidelines (e.g., the Integrated Management of Childhood Illness (IMCI)) [1,2], digital triaging platforms do not rely on health workers referencing algorithm charts and booklets to make their clinical decisions. This makes it easier for new staff to learn the triage process, reducing barriers to implementation [5], and allows for more accurate triaging of children. This is critical to improving outcomes for critically ill children, as the majority of child deaths in health facilities happen within the first 24 to 48 h of admission, and commonly occur as a result of delayed, inadequate, or inappropriate treatment [23]. Consistently, health workers at Jinja Hospital reported being able to use Smart Triage to more easily identify emergency cases, including children who were not obvious emergencies, leading to faster time to treatment and better outcomes for these children. Electronic-based IMCI protocols have previously been implemented in Ghana and Tanzania with similar results [24, 25]. As ICT and digital health platforms become more prevalent in LMICs, the feasibility of implementing electronic-based protocols such as the Smart Triage digital risk prediction algorithm is likely to increase.

Smart Triage also incorporates an automated patient and treatment tracking system, which can be used to identify bottlenecks in patient flow that are unique to the particular environment where the platform is implemented. This can inform QI that targets the entire patient journey within a facility, from triage to treatment. At Jinja, a major delay in time to treatment was occurring after triage and the initial assessment, when a patient needed to travel to the pharmacy or even outside the facility to obtain the treatment. This led to implementation of a drug availability list and emergency drug supply cupboard within the OPD, to reduce delays in time to treatment. In the future, the platform could also be adapted to collect data on the number of children treated for specific diseases weekly and report this data to the pharmacy, to guide procurement and management of specific drugs and address challenges related to resource allocation.

#### Implications for future implementation

Lack of supplies, staffing and training challenges, and staff attitude challenges were the primary factors that reduced feasibility and acceptability. These are common challenges to implementing digital health innovations in low-resource settings, and thus building human and institutional capacity for the safe and appropriate use and scale-up of digital health has been prioritized within national [26] and global [27] digital health strategies. Further, educational tools such as video training modules or training handbooks that remain within each ward could be useful for addressing challenges related to high staff turnover and intake of students. Several study participants also highlighted the potential role of QI champions who could drive change management focused on enhancing the acceptability of Smart Triage as a digital triaging platform for QI. Building local capacity in QI processes and basic data analytics among health workers, hospitals administrators and leadership, and implementing partners (e.g., project staff at non-governmental organizations) could help build a community of practice around data-driven QI, and a network of individuals to provide QI champions with mentorship and training [26]. Publicly recognizing QI teams’ successes, and engaging with community leaders to educate, sensitize, and raise community awareness of new initiatives are also approaches that been used to enhance the acceptability of QI initiatives implemented at health facilities in East Africa, and may address staffing attitude challenges [28]. Demonstrating the effectiveness and need for QI initiatives using data and clear examples has been recommended for improving acceptability among staff who are resistant to adopt new approaches to QI [29]. Engaging both hospital management and frontline health workers can support both ‘top-down’ and ‘bottom-up’ approaches to change management and enhance the success of new data-driven QI initiatives [29].

#### Limitations

A major limitation was that the clinical evaluation of Smart Triage occurred during the COVID-19 pandemic and a COVID-19-related lockdown implemented in Uganda from June to December 2021, leading to staffing shortages at the facility. From November to December 2021, medical interns at Jinja Regional Referral Hospital were participating in a health workers’ strike, exacerbating staffing shortages. Despite less-than-ideal conditions, health workers did report that the platform improved quality of care, suggesting that the platform could have even greater impact under non-pandemic conditions. Additionally, the qualitative study was cross-sectional, and only captured findings within a specific point in time. Further, medical interns and nursing students were not included in the study population. Engaging with health workers of all levels and under non-pandemic and non-striking conditions may help ensure acceptability and sustainability of Smart Triage across all contexts. A further limitation of the study is that caregivers were not included as participants, although we reported health workers’ perceptions of caregivers’ attitudes towards the platform. Caregivers are not the primary users of the platform, so we did not directly assess usability, feasibility, or acceptability among this population. However, caregiver’s willingness to accept the platform is critical to its sustainability, and materials to educate and sensitize caregivers to the platform will be an important component to future implementations.

## Conclusion

The Smart Triage is a digital triaging platform that health workers found easy to use and acceptable with time and experience. This platform could potentially address some of the challenges to implementing current paper-based systems and inform initiatives to improve quality of care for critically ill children at health facilities in low-resource settings. Barriers to the adoption of Smart Triage were identified and need to be considered to ensure effective and sustainable implementation. Our findings, along with those from the ongoing clinical evaluation will inform future implementation and scaling of Smart Triage.

## Electronic supplementary material

Below is the link to the electronic supplementary material.

Supplementary Material 1

Supplementary Material 2

## Data Availability

The datasets generated and analyzed during the current study are not publicly available due to their potentially identifiable nature. They are available from the corresponding author on reasonable request.

## Abbreviations

BLE: Bluetooth Low-Energy.
ETAT: Emergency Triage, Assessment, and Treatment.
ICT: Information and Communications Technology.
IMCI: Integrated Management of Childhood Illness.
LMIC: Low- and Middle-Income Country.
OPD: Outpatient Department.
QI: Quality Improvement.
