# Supplementary material for: Health worker perspectives of Smart Triage, a digital triaging platform for quality improvement at a referral hospital in Uganda: a qualitative analysis
Source: BMC Pediatr. 2022 Oct 13;22:593. doi: 10.1186/s12887-022-03627-1 (PMC9557985; doi:10.1186/s12887-022-03627-1)
Supplement: Supplementary file 1 — Supplementary Material 1 [file 12887_2022_3627_MOESM1_ESM.pdf]

Additional File 1

Table S1: Consolidated criteria for reporting qualitative studies (COREQ): 32-item checklist

No. Item	Guide questions/description	Reported on Page #
Domain 1: Research team and reflexivity		
Personal Characteristics		
1. Interviewer/facilitator	Which author/s conducted the interview or focus group?	Pg 7
2. Credentials	What were the researcher's credentials? E.g. PhD, MD	Pg 7
3. Occupation	What was their occupation at the time of the study?	Pg 7
4. Gender	Was the researcher male or female?	Pg 7
5. Experience and training	What experience or training did the researcher have?	Pg 7
Relationship with participants		
6. Relationship established	Was a relationship established prior to study commencement?	Pg 7 and Additional File 2
7. Participant knowledge of the interviewer	What did the participants know about the researcher? e.g. personal goals, reasons for doing the research	Pg 6, 7 and Additional File 2
8. Interviewer characteristics	What characteristics were reported about the interviewer/facilitator? e.g. Bias, assumptions, reasons and interests in the research topic	Pg 7
Domain 2: study design		
Theoretical framework		

9. Methodological orientation and Theory	What methodological orientation was stated to underpin the study? e.g. grounded theory, discourse analysis, ethnography, phenomenology, content analysis	Pg 7
Participant selection		
10. Sampling	How were participants selected? e.g. purposive, convenience, consecutive, snowball	Pg 6
11. Method of approach	How were participants approached? e.g. face-to-face, telephone, mail, email	Pg 7
12. Sample size	How many participants were in the study?	Pg 6
13. Non-participation	How many people refused to participate or dropped out? Reasons?	Pg 8
Setting		
14. Setting of data collection	Where was the data collected? e.g. home, clinic, workplace	Pg 7
15. Presence of non-participants	Was anyone else present besides the participants and researchers?	Pg 7
16. Description of sample	What are the important characteristics of the sample? e.g. demographic data, date	Pg 8
Data collection		
17. Interview guide	Were questions, prompts, guides provided by the authors? Was it pilot tested?	Additional File 2
18. Repeat interviews	Were repeat interviews carried out? If yes, how many?	Pg 7
19. Audio/visual recording	Did the research use audio or visual recording to collect the data?	Pg 7
20. Field notes	Were field notes made during and/or after the interview or focus group?	Pg 7
21. Duration	What was the duration of the interviews or focus group?	Pg 7

22. Data saturation	Was data saturation discussed?	Pg 6
23. Transcripts returned	Were transcripts returned to participants for comment and/or correction?	N/A
Domain 3: analysis and findings		
Data analysis		
24. Number of data coders	How many data coders coded the data?	Pg 7
25. Description of the coding tree	Did authors provide a description of the coding tree?	Pg 8
26. Derivation of themes	Were themes identified in advance or derived from the data?	Pg 7
27. Software	What software, if applicable, was used to manage the data?	Pg 7
28. Participant checking	Did participants provide feedback on the findings?	N/A
Reporting		
29. Quotations presented	Were participant quotations presented to illustrate the themes/findings? Was each quotation identified? e.g. participant number	Yes, see pg 9-15
30. Data and findings consistent	Was there consistency between the data presented and the findings?	Yes, see pg 8-15
31. Clarity of major themes	Were major themes clearly presented in the findings?	Yes, see pg 8-15
32. Clarity of minor themes	Is there a description of diverse cases or discussion of minor themes?	Yes, see pg 8-15
