# Supplementary material for: Health worker perspectives of Smart Triage, a digital triaging platform for quality improvement at a referral hospital in Uganda: a qualitative analysis
Source: BMC Pediatr. 2022 Oct 13;22:593. doi: 10.1186/s12887-022-03627-1 (PMC9557985; doi:10.1186/s12887-022-03627-1)
Supplement: Supplementary file 2 — Supplementary Material 2 [file 12887_2022_3627_MOESM2_ESM.pdf]

Additional File 2

Table S2: Interview guide for assessing health worker perspectives of Smart Triage

**Administrative information – Filled out by participant**	
**ID number:**	
**Date of informed consent form signed:** |D|D| - |M|M|M| - |Y|Y|Y|Y|	
**First Name,**	**Sex:** ☐ Female ☐ Male ☐ Prefer not to say
**Last name,**	**Age group: <20, 20-30, 30-40, 40-50, 50+**
**Program and year graduated**	
**Total months/years worked in current field**	|M|M| - |Y|Y|
**Total months/years worked at current hospital**	|M|M| - |Y|Y|
**Current department**	
**Current position**	
**Administrative information – Filled out by researcher**	
**Interview Group number (if applicable)**	
**Does the participant agree to be audio recorded?** ☐ Yes ☐ No<br>(If no, participant will be excluded from the study)	
**Name of interviewer:**	
**Date of interview:** |D|D| - |M|M|M| - |Y|Y|Y|Y|	
**Location of interview:**<br>☐ **Jinja Regional Referral Hospital**<br>☐ **Other:** ____________________________	
**Interview start time:** |H|H| : |M|M| *military time*<br>**Interview end time:** |H|H| : |M|M| *military time*	

<u>Instructions for qualitative research staff</u>:

- Use this document as a guide to conduct the interviews with the **hospital staff (HS)**.
- Conduct the interview in the language with which the HS feels most comfortable.
- The interview should take place in a quiet place that allows privacy.
- Please introduce each question separately. The interview should flow as a conversation. If you notice that the HS is hesitant in answering, does not give an in-depth response, or the response is not satisfactory, please probe or ask follow-up questions, but do NOT prompt any specific answer. Several probes are suggested, and you may also ask follow-up questions that are not listed in this guide but are necessary for the complete expression of the HS's views.
- Please **record the interview** using the designated audio recorder and state the HS ID number at the beginning and end of the recording.
- All comments from the HS should be recorded.
- All responses must be kept confidential. Do not discuss or share responses with anyone outside of the PRST study team.

**Interview Script**
Example introduction:<br>*"Welcome, my name is <interviewer>. I would like to thank you for participating in this interview today. I'm here today to ask you questions and hear your thoughts, opinions, and feelings about the digital triage tool that has been operational at your facility for the past 7 months. <Interviewer will provide a brief description of the tool and events to clarify the subject of the interview>. I'll begin by asking you a few questions about yourself and your role at this hospital…"*
**A. Demographic Information**
1. First, we will start with some questions about yourself;<br>2. What is your first and last name?<br>3. How many years of education and training have you completed?<br>4. What is your highest level of education completed?
**B. Current role at this facility**
1. How long have you been employed at this healthcare facility?<br>2. What is your current job title or current role at this facility?<br>3. How long have you been in this role at this facility?<br>4. What are your responsibilities in this role?<br>*Probes: What does a typical day look like to you?*<br>5. Can you please explain your experience with technology? What about outside your profession? Have you previously used technology to identify or treat patients in your previous jobs?
**C. Smart Triage**
1. What aspects of Smart Triage have you used? Can you briefly tell me more about them/it?<br>2. Tell me about the training you received to prepare you for using Smart Triage. Who gave the training?<br>3. Do you feel you were properly trained to use Smart Triage?<br>*Probes: What are your thoughts in regards to the length of training? If they think the training was too long/short - what are your thoughts in regards to the method of delivery for the training?*<br>4. Which aspects of using Smart Triage were easy to learn? Which aspects were difficult? And why?<br>*Probes: What barriers did you anticipate/experience when using this tool?*<br>5. What do you like (if anything) about Smart Triage overall? What do you dislike (if anything)?<br>*Probe: How do you find using this tool?*<br>6. What feedback have you gotten from the patients and their caregivers? What about other hospital staff?

Probe: What parts of the platform do patients/ caregivers ask questions about? Are there parts that they like? Dislike? Do they accept being seen in a different order than they arrive? Do they trust the triage category given to them?

7. What do you think about using Bluetooth technology to track patient movement?
   Probe: Do you have specific concerns about Bluetooth technology? Have you heard others express concerns about Bluetooth technology? Do you use Bluetooth technology in your personal life? Description of the technology "Bluetooth is a wireless technology standard used for exchanging data between fixed and mobile devices over short distances using short-wavelength UHF radio

8. Would you consider triage categorization given by the platform accurate? Why or why not?
   Probe: How does is compare to the previous process (first-come, first serve)? Do the risk algorithm identify those who are most in need of care?

9. What information does Smart Triage currently provide that is of interest and of use to you?
   Probes: Is there information is not giving you that you want? Is there information it currently gives that is not useful?

10. How has Smart Triage impacted patient care at your facility?
    Probe: Has it changed how much time you spend with each patient? How quickly patients receive treatment?

11. What is the biggest change Smart Triage has had at your facility? In your work?
    Probe: Improved patient care? Caregiver satisfaction? Staff satisfaction?

12. How can use of the Smart Triage app be improved?

13. Would health workers in other hospitals would like this kind of technology?

14. Can you describe your experience with quality improvement (or "QI") initiatives linked to Smart Triage?
    Probe: Have you attended training sessions on QI? What role did you play in these initiatives? What was the main goal of these initiatives? If no experience – what prevented you from taking part in these initiatives?

    If they have no experience at all, skip to question 19.

15. How do you feel these initiatives affected the standard of care at your facility?
    Probe: Did it impact how patients were treated? Did it change outcomes for patients? Why or why not?

16. What did you enjoy about the training or initiatives at your facility?
    Probe: Were the sessions interactive? Was the information useful? Did it impact your work positively?

17. How could QI training or initiatives could be handled differently in the future at your facility? At other facilities?
    Probe: What was missing? What else do you wish you learned? What would have helped you learn more or have a greater impact at your facility?

18. How did your experience using Smart Triage for QI compare to other QI initiatives you have been involved in? *Probe: Does it make implementing changes easier or harder? How?*

19. Do you have any other comments about Smart Triage or QI training that we did not talk about?
